# Phloretin suppresses neuroinflammation by autophagy-mediated Nrf2 activation in macrophages

**DOI:** 10.1186/s12974-021-02194-z

**Published:** 2021-07-04

**Authors:** Tess Dierckx, Mansour Haidar, Elien Grajchen, Elien Wouters, Sam Vanherle, Melanie Loix, Annick Boeykens, Dany Bylemans, Kévin Hardonnière, Saadia Kerdine-Römer, Jeroen F. J. Bogie, Jerome J. A. Hendriks

**Affiliations:** 1 Department of Immunology and Infection, Biomedical Research Institute, Hasselt University, Diepenbeek, Belgium; 2 Department of Chemistry, Odisee University College, Gent, Belgium; 3 Research Station for Fruit Cultivation, Sint-Truiden, Belgium; 4 Department of Biosystems, KU Leuven, Heverlee, Belgium; 5 Inflammation, Microbiome and Immunosurveillance, INSERM UMR996, Université Paris-Saclay, Châtenay-Malabry, France

**Keywords:** Phloretin, Macrophages, Neuroinflammation, Multiple sclerosis, Autoimmunity

## Abstract

**Background:**

Macrophages play a dual role in neuroinflammatory disorders such as multiple sclerosis (MS). They are involved in lesion onset and progression but can also promote the resolution of inflammation and repair of damaged tissue. In this study, we investigate if and how phloretin, a flavonoid abundantly present in apples and strawberries, lowers the inflammatory phenotype of macrophages and suppresses neuroinflammation.

**Methods:**

Transcriptional changes in mouse bone marrow-derived macrophages upon phloretin exposure were assessed by bulk RNA sequencing. Underlying pathways related to inflammation, oxidative stress response and autophagy were validated by quantitative PCR, fluorescent and absorbance assays, nuclear factor erythroid 2–related factor 2 (Nrf2) knockout mice, western blot, and immunofluorescence. The experimental autoimmune encephalomyelitis (EAE) model was used to study the impact of phloretin on neuroinflammation in vivo and confirm underlying mechanisms.

**Results:**

We show that phloretin reduces the inflammatory phenotype of macrophages and markedly suppresses neuroinflammation in EAE. Phloretin mediates its effect by activating the Nrf2 signaling pathway. Nrf2 activation was attributed to 5′ AMP-activated protein kinase (AMPK)-dependent activation of autophagy and subsequent kelch-like ECH-associated protein 1 (Keap1) degradation.

**Conclusions:**

This study opens future perspectives for phloretin as a therapeutic strategy for neuroinflammatory disorders such as MS.

**Trial registration:**

Not applicable.

**Supplementary Information:**

The online version contains supplementary material available at 10.1186/s12974-021-02194-z.

## Background

Active multiple sclerosis (MS) lesions are characterized by the presence of numerous macrophages [1–5]. Early studies demonstrated that macrophages adopt a pro-inflammatory phenotype in MS lesions, thereby promoting neuroinflammation, demyelination and neurodegeneration. Disease-promoting effector functions include the presentation of central nervous system (CNS)-derived antigens to autoreactive T cells and the production of inflammatory mediators such as pro-inflammatory cytokines, reactive oxygen species (ROS) and nitric oxide (NO) [6, 7]. More recently, it was found that macrophages also have beneficial functions in MS lesions as they can reshape their phenotype to one that is typically associated with immunosuppression and CNS repair. This protective phenotype is characterized by a reduced production of pro-inflammatory mediators, production of anti-inflammatory mediators and growth factors, and activation of anti-oxidative pathways such as the nuclear factor erythroid 2–related factor 2 (Nrf2) pathway [8–14]. Since the inflammatory status of macrophages has been linked to the progression of MS and the development of chronic active lesions, driving macrophages to a beneficial phenotype is considered to be a promising strategy to limit the progression of MS [15].

Dietary components drive macrophage function and neuroinflammation [16]. In particular, the family of flavonoids is increasingly being acknowledged to contain promising compounds that influence pathogenic pathways and modulate the phenotype of immune cells such as macrophages [17, 18]. Flavonoids form one of the largest phytonutrient families that contains over 8000 phenolic compounds with diverse bioactivity. Several members of the flavonoid family display anti-inflammatory and anti-oxidative effects on macrophages [19–21]. The flavonoid phloretin is a member of the dihydrochalcones and is present in commonly consumed fruits such as apples and strawberries. Phloretin is known to exert immunomodulatory features and is widely used for skincare due to its anti-oxidative characteristic [22–24]. Moreover, phloretin is a glucose transporter (GLUT) inhibitor, a feature that affects the phenotype of macrophages since macrophage activation is fueled by GLUT [25, 26]. Overall, these characteristics make phloretin a promising compound to modulate the phenotype of macrophages and neuroinflammation.

In this study, we show that phloretin drives macrophages towards a less-inflammatory phenotype and alleviates neuroinflammation in the experimental autoimmune encephalomyelitis (EAE) model. RNA sequencing and functional experiments identified the Nrf2 pathway as a hub and driver of this protective macrophage phenotype induced by phloretin. AMPK-dependent activation of the autophagy machinery and subsequent SQSTM1/p62-mediated (hereafter referred to as p62) degradation of Keap1, an adaptor that facilitates proteasomal Nrf2 degradation, was found to underlie Nrf2 activation in macrophages. These findings show that phloretin has the potential to be used in therapeutic strategies for neuroinflammatory disorders.

## Methods

### Antibodies and chemical reagents

Phloretin (Sigma Aldrich) was dissolved in 50 mM KOH to a 15 mM stock solution and stored at − 20 °C. Further dilutions were made in a RPMI1640 (Gibco) medium. For in vivo treatment, phloretin was dissolved in 1 N NaOH, whereafter the pH was readjusted to 7.2 with 1 N HCL, and the solution further diluted in physiological water to obtain a concentration of 50 mg/kg. BML-275 (1 μM, Santa Cruz Biotechnology) was added 1 h before phloretin treatment to inhibit AMPK activation. Bafilomycin A1 (baf, 0.1 μM, InvivoGen) was added 2 h before collection to block the fusion of autophagosomes and lysosomes. Lipopolysaccharide (LPS, 100 ng/ml, Sigma-Aldrich) was used to stimulate cells for inflammatory phenotyping. Phorbol 12-myristate 13-acetate (PMA, 100 ng/ml, Sigma-Aldrich) was used to induce ROS production. The following antibodies were used for western blot: mouse anti-β-actin (1:10,000; sc-47778, Santa Cruz Biotechnology), mouse anti-GAPDH (1:10 000; AB_2537659, Invitrogen), rabbit anti-AMPK (1:1000; 5831S, Cell Signaling Technology), rabbit anti-phosphorylated AMPK (1:1000; 2535S, Cell Signaling Technology), rabbit anti-LC3 (1:1000; L7543, Sigma-Aldrich), rabbit anti-p62 (1:1000; 23214, Cell Signaling Technology). The following antibodies were used for immunofluorescence: rat anti-CD3 (1:150; MCA500G, Bio-Rad), rat anti-F4/80 (1:100; MCA497G, Bio-Rad), rabbit anti-LC3 (1:1000; L7543, Sigma-Aldrich), rabbit anti-p62 (1:500; 23214, Cell Signaling Technology), rabbit anti-Keap1 (1:500; 60027-1-Ig, Proteintech Europa), rabbit anti-TMEM119 (1:100, ab209064, Abcam). Appropriate secondary antibodies were purchased from Invitrogen.

### Mice

Wild-type (WT) C57BL/6JOlaHsd mice were purchased from Envigo. Animals were fed a regular diet and housed in the animal facility of the Biomedical Research Institute of Hasselt University. All experiments were performed according to institutional guidelines and were approved by the ethical committee for animal experiments of Hasselt University.

### Cell culture

Bone marrow-derived macrophages (BMDMs) were isolated from WT and Nrf2 knockout (KO) C57BL/6JOlaHsd mice, purchased from Envigo and provided by the RIKEN BRC according to an MTA to Prof S. Kerdine-Römer respectively [27, 28]. BMDMs were obtained as described previously [29]. In short*,* tibial and femoral bone marrow cells from 12-week-old WT and Nrf2 KO C57BL/6JOlaHsd mice were cultured in 10-cm petri plates at a concentration of 10 × 10^6^ cells/plate, in RPMI1640 medium supplemented with 10 % foetal calf serum (FCS, Gibco), 50 U/ml penicillin (Invitrogen), 50 U/ml streptomycin (Invitrogen), and 15 % L929-conditioned medium (LCM). After differentiation, BMDMs were detached at 37 °C with 10 mM EDTA in PBS (Gibco) and cultured (0.5 × 10^6^ cells/ml) in RPMI1640 supplemented with 10 % FCS, 50 U/ml penicillin, 50 U/ml streptomycin and 5 % LCM (37 °C, 5 % CO_2_). Microglia cultures were isolated from brains of postnatal P1–3 *C57BL/6JOlaHsd* pups. After the brain stem, choroid plexus and meninges were removed, the brains were mechanically dissociated and enzymatically digested for 15 min with 1× trypsin (Gibco) at 37 °C. Afterwards, the cell suspension was seeded out in DMEM a high-glucose medium (Sigma) supplemented with 30% LCM, 10% FCS, 50 U/ml penicillin and 50 U/ml streptomycin in T75 culture flasks. Two to 3 days later, a complete medium change was performed. Mixed glial cultures were shaken (230 rpm, 3 h, 37 °C) after 6–7 days to obtain pure microglia cultures.

### RNA sequencing

Cells were pretreated with phloretin (50 μM) for 20 h and LPS-stimulated (100 ng/ml) for 6 h. Cell lysis was performed using Qiazol Lysis reagent (Qiagen). RNA was extracted from cells using the RNeasy mini kit (Qiagen). Samples were then processed by the Genomics Core Leuven (Belgium). Library preparation was performed with the Lexogen’s QuantSeq kit to generate Illumina compatible libraries. Libraries were sequenced on the Illumina HiSeq4000 sequencing system. Splice-aware alignment was performed with STAR v2.6.1b [30]. Quantification of reads per gene was performed with HT-seq Count v2.7.14. Count-based differential expression analysis was done with R-based (The R Foundation for Statistical Computing, Vienna, Austria) Bioconductor package DESeq2. A list of differentially expressed genes was selected at a p < 0.05 and used as an input for the core analysis by QIAGEN’s ingenuity pathway analysis (IPA). All RNA sequencing (RNA-seq) data discussed in this publication have been deposited in NCBI’s Gene Expression Omnibus (Edgar et al., 2002) and are accessible through GEO Series accession number GSE166608.

### Quantitative reverse transcription PCR

Cells were pretreated with phloretin (50 μM) for 20 h and LPS stimulated (100 ng/ml) for 6 h. Lysis was performed by using Qiazol Lysis reagent (Qiagen). RNA was extracted using the RNeasy mini kit (Qiagen). RNA concentration and quality were determined with a Nanodrop spectrophotometer (Isogen Life Science). cDNA synthesis was conducted using the Quanta qScript cDNA SuperMix (Quanta Biosciences) per the manufacturer’s instructions. qPCR was performed on a StepOnePlus™ Real-Time PCR system (Applied biosystems) using a SYBR green mix containing 1× SYBR green (Applied Biosystems), 0.3 μM primers (Integrated DNA Technologies), 12.5 ng cDNA and nuclease-free water. The comparative Ct method was used to quantify gene expression. Data were normalized to the most stable reference genes cyclin A and hypoxanthine phosphoribosyltransferase 1. Primer sequences are available on request.

### Determination of reactive oxygen species

Cells were pretreated with phloretin (50 μM) for 2 h. Afterwards, cells were stimulated with PMA (15 min, 100 ng/ml) and ROS production was measured using the fluorescent probe 2′,7′-dichlorodihydrofluorescein diacetate at 10 μM in PBS for 30 min. Fluorescence was measured using the fluorescence FLUOstar optima microplate reader (BMG Labtech, Ortenberg, Germany) (excitation: 495 nm, emission: 529 nm).

### Measurements of nitric oxide

Cells were pretreated with phloretin (50 μM) for 2 h. Afterwards, cells were stimulated with LPS (24 h, 100 ng/ml). NO was indirectly monitored using the Griess reagent nitrite measurement kit (Abcam). Briefly, nitrite reacts with sulphanilamide and N-(1-naphthyl)ethylenediamine dihydrochloride to produce a pink azo dye. Absorbance of this azo derivative was then measured at 540 nM using a microplate reader (iMark, Bio-Rad).

### Western blot

Cells were treated with phloretin (50 μM) and LPS (100 ng/ml) for 1 h or 24 h to determine AMPK activation or p62, LC3 and Keap1 protein levels respectively. Cells were lysed using RIPA-buffer (150 mM NaCl, 1 % Triton X-100, 0.5 % sodium deoxycholate, 1 % SDS, 50 mM Tris), and separated via sodium dodecyl sulphate polyacrylamide gel electrophoresis. Gels were transferred to a PVDF-membrane (VWR) and blots were blocked for 1 h in 5 % bovine serum albumin in Tris-buffered saline containing 0.1 % Tween-20 (TBS-T). Membranes were probed with primary antibodies overnight at 4 °C, washed with TBS-T and incubated with the corresponding secondary horseradish peroxidase-labeled antibody for 1 h at room temperature (RT). Immunoreactive signals were detected with enhanced chemiluminescence (ECL Plus, Thermo Fisher) using the Amersham Imager 680 (GE Healthcare Life Sciences). The densities of the bands were determined using ImageJ.

### Immunofluorescence

Spinal cord cryosections were air-dried and fixed in ice-cold acetone for 10 min at − 20 °C. Mouse BMDMs were cultured on glass cover slides and fixed in ice-cold methanol for 10 min at − 20 °C. Sections and BMDMs were blocked for 30 min using Dako protein block (Agilent). Afterwards, they were incubated overnight at 4 °C with primary antibodies, washed and incubated with the appropriate secondary antibodies for 1 h at RT. Images of the spinal cord tissue were taken using a Nikon Eclipse 80i microscope (10× objective) and NIS Elements BR 3.10 software (Nikon). Images of BMDMs stained for p62, LC3 and Keap1 were taken using the Zeiss LSM 880 confocal microscope and were Airyscan corrected (63× objective). P62-and LC3-positive puncta were determined by semi-automated puncta analysis imageJ. In short, after the image was made binary and cells were selected by hand, puncta were analysed per cell. P62 and Keap1 colocalization was performed using a colocalization pipeline in the CellProfiler software [31]. The images shown in the figures are digitally enhanced.

### Experimental autoimmune encephalomyelitis model

Eleven-week-old C57BL/6JOlaHsd mice were immunized subcutaneously with 200 ng of myelin oligodendrocyte glycoprotein peptide (MOG35−55) emulsified in 100 μl complete Freund’s supplemented with 4 mg/ml of *Mycobacterium tuberculosis* (EK2110, Hooke Laboratories). Immediately after MOG immunization and after 24 h, mice were intraperitoneally injected with 50 ng pertussis toxin (EK2110 kit, Hooke Laboratories) to induce EAE. EAE animals were treated daily with phloretin or vehicle (50 mg/kg, intraperitoneal (ip)) after 6 days of immunization (prophylactic setup) or after disease onset (clinical score > 1, therapeutic setup). Mice were weighed and scored daily for neurological signs of the disease according to the manufacturer’s mouse EAE scoring guide: 0: no clinical symptoms, 0.5: tip of tail is limp, 1: limp tail, 1.5: limp tail and hind leg inhibition 2: limp tail and weakness of hind legs, 2.5: limp tail and dragging of hind legs, 3: limp tail and complete paralysis of the hind legs, 3.5: limp tail and complete paralysis of hind legs and mouse is unable to right itself when placed on its side, 4: paralysis to the diaphragm, 5: death by EAE.

### Statistical analysis

GraphPad Prism was used to statistically analyse the data, which are represented as mean ± s.e.m. D’Agostino and Pearson omnibus normality test was used to test for normal distribution. Two-tailed unpaired Student’s t-test (with Welch’s correction if necessary) was used for normally distributed data. The Mann-Whitney analysis was used for data that did not pass the normality test. p-values < 0.05 were considered to demonstrate significant differences (*p < 0.05, **p < 0.01, ***p < 0.001 and ****p < 0.0001).

## Results

### Transcriptional changes associated with phloretin treatment of macrophages

**Fig. 1:**
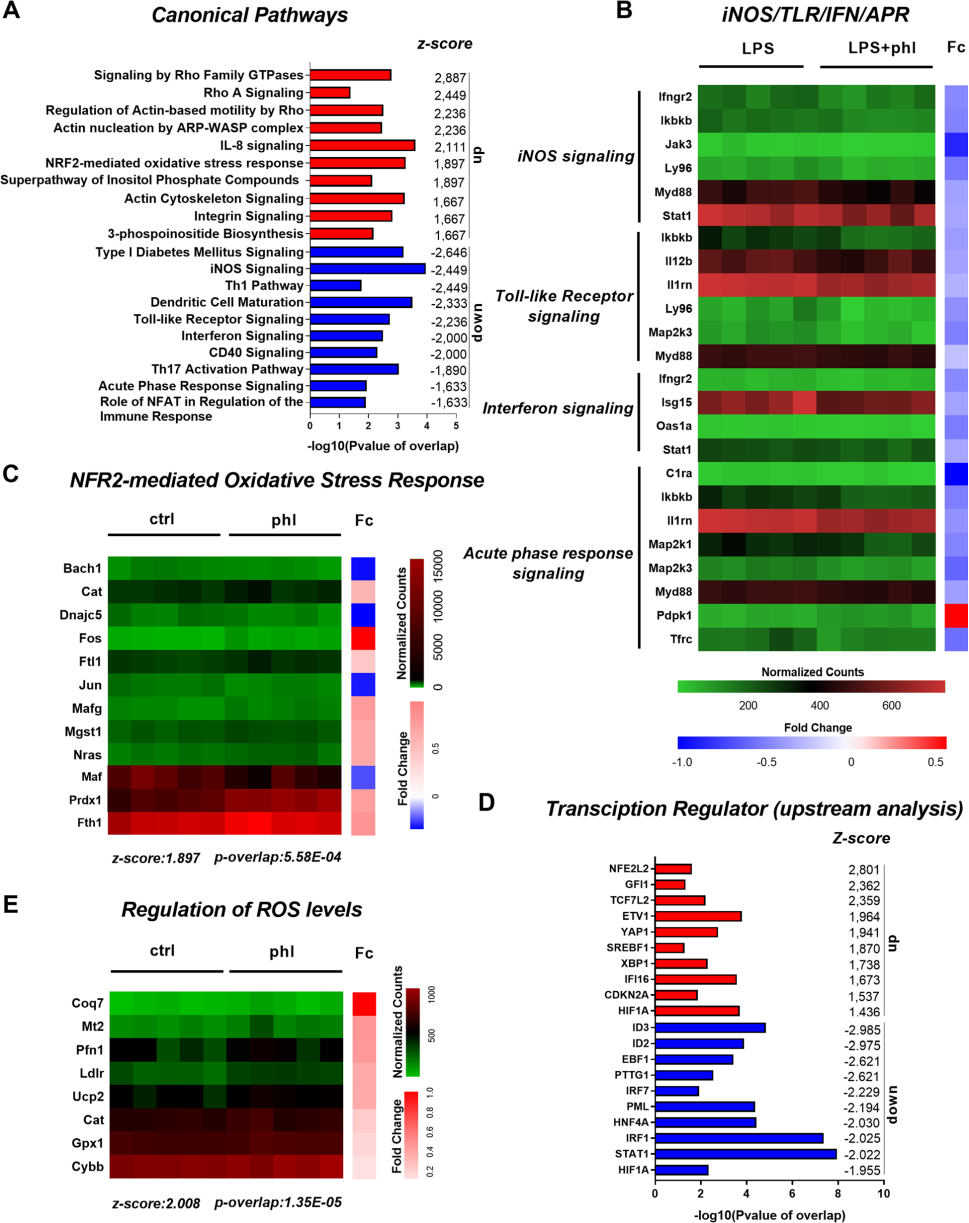
Transcriptional changes associated with phloretin treatment of macrophages. RNA sequencing was performed to establish the anti-inflammatory effects of phloretin and identify the underlying mechanisms. Differentially expressed genes were used as input for the core analysis in ingenuity pathway analysis (IPA) (n = 5, cut-off criteria p < 0.05, see supplementary Fig. 1). **A** Pathway analysis showing down- and upregulated canonical pathways of phloretin-stimulated macrophages treated with or without LPS respectively. -Log (P-value of overlap) and down- or upregulated canonical pathways with corresponding z-score are indicated at x- and y-axis, respectively. **B**, **C** Heat map representing the normalized counts of differentially expressed genes associated to the pro-inflammatory canonical pathways (iNOS-, toll-like receptor-, acute phase response- and interferon-signaling) and the Nrf2 pathway. A colour gradient was used to indicate the normalized counts and corresponding fold change (Fc) differences per sample and gene, respectively. **D** Upstream analysis showing down- and upregulated transcription regulators of phloretin-stimulated macrophages treated with or without LPS respectively. **E** Downstream analysis of the RNA-seq samples in IPA illustrated that phloretin upregulated the expression of a set of genes involved in the regulation of ROS levels as one of the main downstream functional effects (z-score: 2.008). Ctrl, control; phl, phloretin; Fc, Fold change

To establish potential anti-inflammatory effects and identify underlying mechanisms of phloretin treatment on macrophages, we performed bulk RNA sequencing (supplementary Fig. 1). Pathway analysis of activated macrophages treated with phloretin showed that differentially expressed genes were overrepresented in canonical pathways related to inflammation, such as iNOS (z-score: − 2.449), toll-like receptor (z-score: − 2.236), interferon signaling (z-score: − 2) and acute phase response pathway (z-score: − 1.633) (Fig. 1A, B). Similar to canonical pathway analysis, upstream analysis of the RNA-seq data predicted that phloretin reduced the activation of key pro-inflammatory transcription regulators such as IRF7 (z-score: − 2.229), IRF1 (z-score: − 2.025) and STAT1 (z-score: − 2.022) (Fig. 1D). Next to downregulating macrophage pro-inflammatory pathways, RNA-seq analysis of phloretin-treated macrophages showed that, amongst other pathways, phloretin potently activated the Nrf2 pathway (z-score: 1.897), evidenced by upregulation of Nrf2-associated genes such as *mafG* and *prdx1* (Fig. 1A, C). Even more, Nrf2 (NFE2L2*)* was identified as the most activated upstream transcriptional regulator (z-score: 2.801) and regulation of ROS levels was identified as one of the most upregulated biological functions in phloretin-treated BMDMs (z-score: 2.008) (Fig. 1D, E). Collectively, findings show that phloretin activates Nrf2 and suppresses the inflammatory phenotype of macrophages.

### The Nrf2 pathway controls the phenotype of phloretin-treated macrophages

**Fig. 2:**
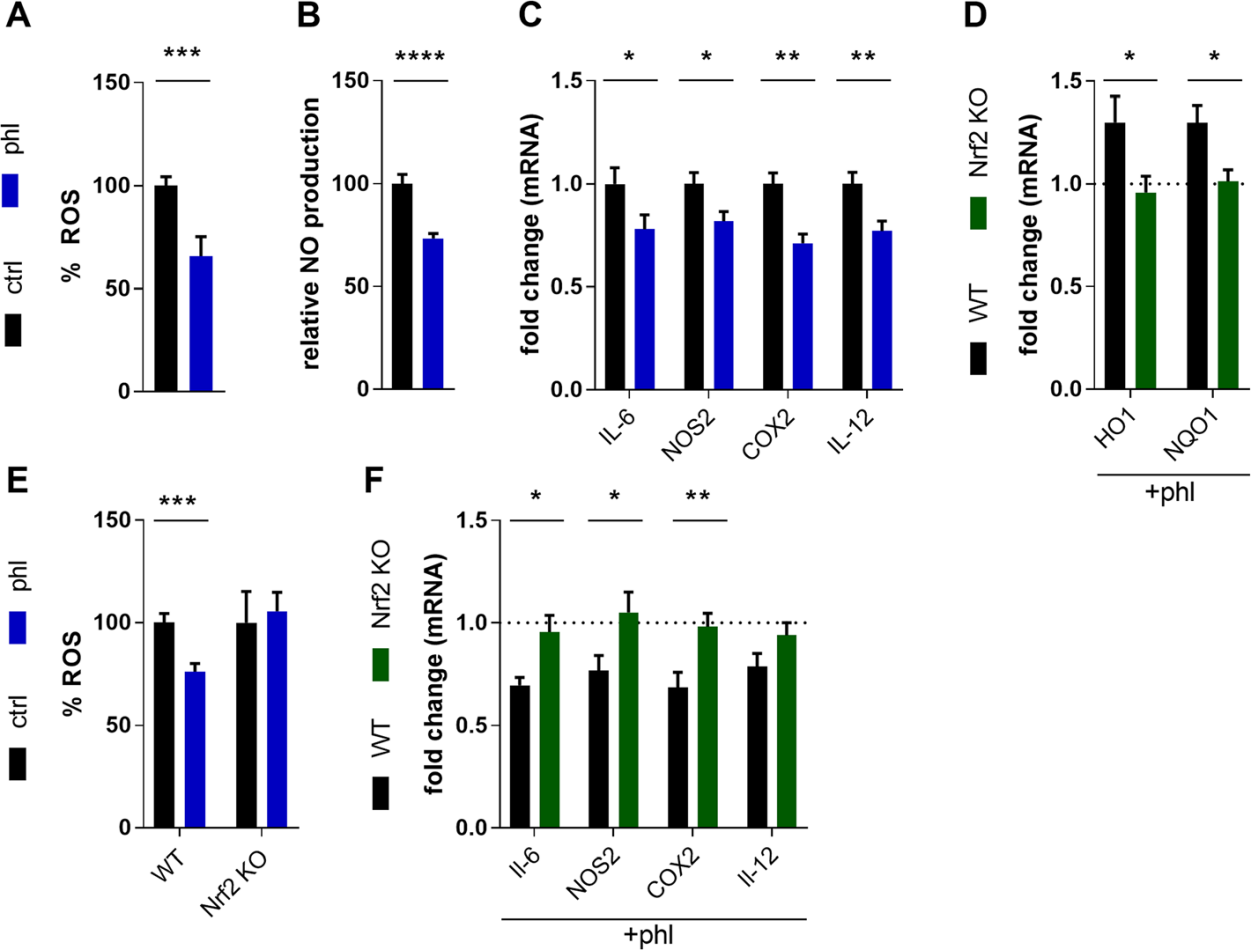
The Nrf2 pathway controls the phenotype of phloretin-treated macrophages. **A** ROS production in vehicle- or phloretin-treated BMDMs stimulated with PMA (n = 9). **B** NO production in vehicle- or phloretin-treated BMDMs stimulated with LPS (n = 9–11) **C.** mRNA levels of the pro-inflammatory genes *IL-6*, *NOS2*, *COX2* and *IL-12* in vehicle- or phloretin-treated BMDMs stimulated with LPS (n = 13–16). **D** mRNA levels of Nrf2-response genes *HO1* and *NQO1* in LPS-stimulated WT and *Nrf2* KO BMDMs (n = 9–10). The dotted line represents corresponding LPS-stimulated control cells stimulated without phloretin. **E** ROS production (n = 5–9) in vehicle- or phloretin-treated WT and *Nrf2* KO BMDMs after PMA stimulation. **F** Pro-inflammatory gene expression of *NOS2*, *IL-6*, *COX2* and *IL-12* (n = 9–10) in phloretin-treated WT and *Nrf2* KO BMDMs after LPS stimulation. The dotted line represents corresponding LPS-stimulated control cells stimulated without phloretin. Ctrl, control; phl, phloretin. Data are represented as mean ± s.e.m. *p < 0.05, **p < 0.01, ***p < 0.001 and ****p < 0.0001

Next, we validated the anti-inflammatory effect of phloretin and determined whether it modulates the inflammatory phenotype of macrophages by acting on Nrf2. Reduced ROS levels were observed in phloretin-treated WT BMDMs stimulated with PMA (Fig. 2A). Moreover, reduced NO production and mRNA levels of pro-inflammatory genes *NOS2*, *IL-6*, *COX2* and *IL-12* were observed in phloretin-treated WT BMDMs stimulated with LPS (Fig. 2B,C). In relation to the crucial role of Nrf2 in inducing a less-inflammatory phenotype, our data show that phloretin activates Nrf2-response genes *HO1* and *NQO1* in WT but not *Nrf2* KO BMDMs stimulated with LPS (Fig. 2D). Furthermore, phloretin treatment reduced ROS production in WT but not in *Nrf2* KO BMDMs (Fig. 2E). Aside from controlling anti-oxidative responses, Nrf2 is reported to suppress the inflammatory phenotype of macrophages [12]. In support of the latter, phloretin was unable to reduce the expression of pro-inflammatory genes *NOS2, IL-6, COX2* and *IL-12* in activated *Nrf2-*deficient BMDMs (Fig. 2F). Altogether, these results indicate that Nrf2 drives the phloretin-mediated inflammatory phenotype shift of macrophages.

### Phloretin promotes AMPK activation

**Fig. 3:**
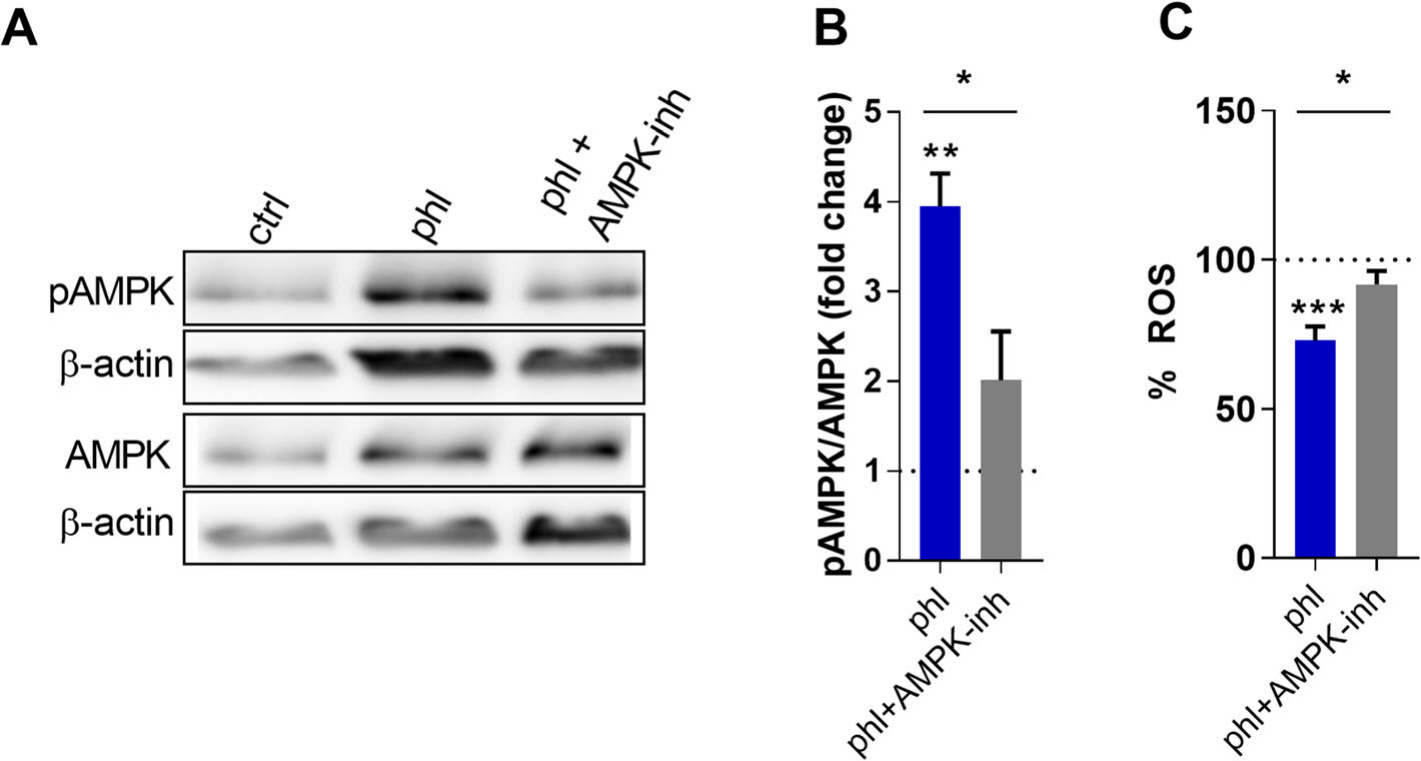
Phloretin activates AMPK. **A**, **B** Western blot quantification and representative bands of pAMPK and AMPK in LPS-activated BMDMs stimulated with phloretin or phloretin and the AMPK inhibitor BML-275 together (n = 3). The dotted line represents control cells stimulated with LPS. **C** ROS production in phloretin-treated or phloretin and AMPK inhibitor-treated BMDMs (n = 10). The dotted line represents control cells stimulated with PMA. Ctrl, control; phl, phloretin. Data are represented as mean ± s.e.m. *p < 0.05, **p < 0.01 and ***p < 0.001

Phloretin is a well-defined GLUT inhibitor and recent studies highlight the importance GLUT-mediated glucose uptake for macrophage activation [26, 32]. Hence, we determined whether phloretin could activate the energy sensor AMPK, which is activated upon low energy/glucose levels. Our results show that phloretin treatment leads to AMPK phosphorylation and activation (Fig. 3A, B). Moreover, the addition of the AMPK inhibitor BML-275 largely prevents AMPK activation in phloretin-treated BMDMs (Fig. 3A, B). Even more, our findings demonstrate that AMPK activation is essential for phloretin to suppress ROS production, as shown by higher ROS levels in BMDMs treated with both phloretin and AMPK inhibitor compared to BMDMs treated with phloretin alone (Fig. 3C). In total, our data suggest that phloretin-induced AMPK activation is crucial for driving macrophages towards a less-inflammatory phenotype.

### Phloretin stimulates autophagy in an AMPK-dependent manner

**Fig. 4:**
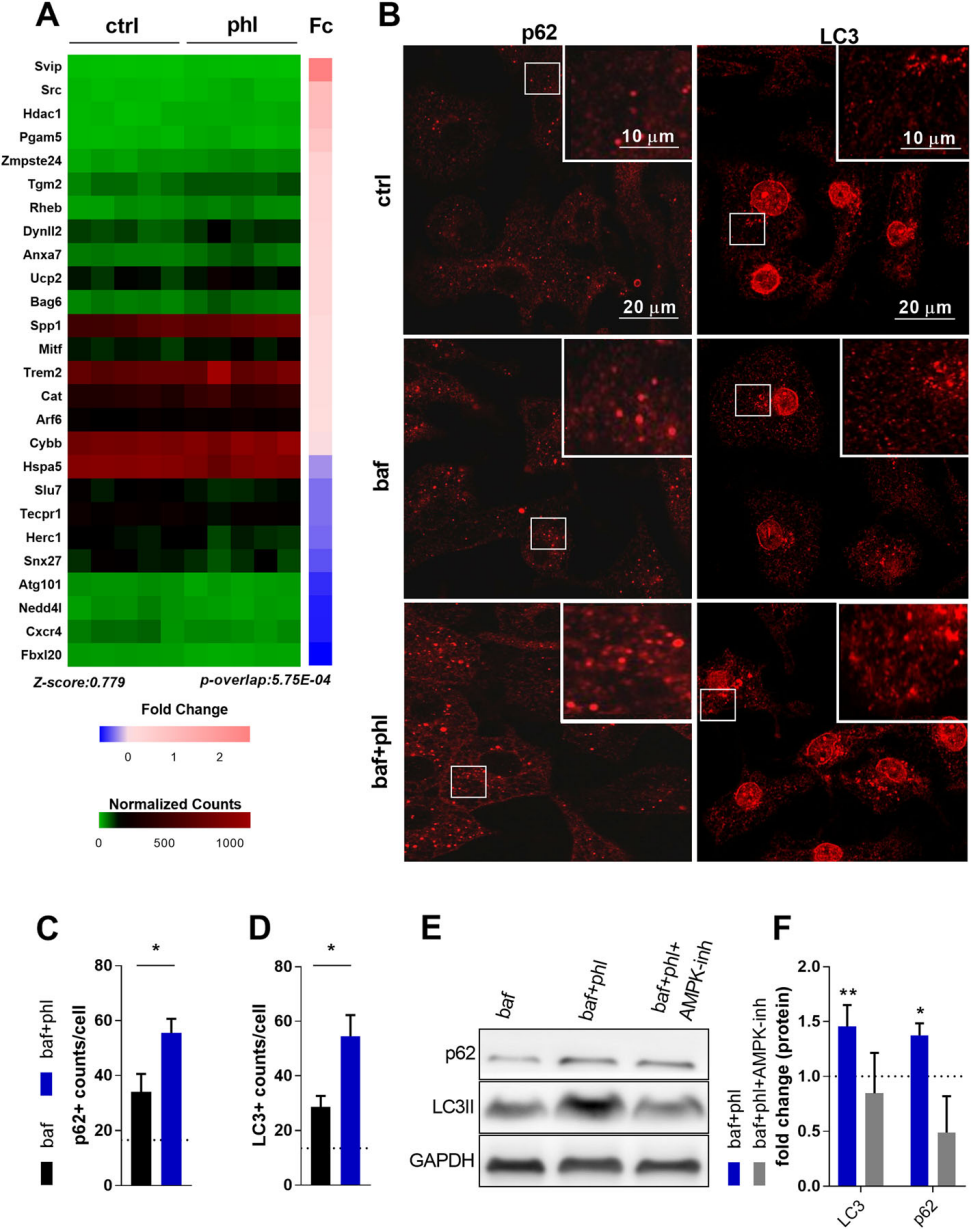
Phloretin stimulates autophagy in an AMPK-dependent manner. **A** Downstream analysis of the RNA-seq data obtained from phloretin treated BMDMs stimulated with LPS illustrated autophagy as one of the downstream biological processes that is activated by phloretin (z-score: 0.779). Data are represented by a heat map containing the normalized counts of genes associated with autophagy. A colour gradient was used to indicate the normalized counts and corresponding Fold change (Fc) differences, per sample and gene respectively. **B–D** Quantification and representative images of LC3 and p62 staining in phloretin-treated BMDMs stimulated with bafilomycin A1 (n = 5). The dotted line represents untreated cells (controls). **E**, **F** Western blot quantification and representative bands of the autophagy markers LC3II and p62 in phloretin-treated BMDMs stimulated with bafilomycin A1 alone or bafilomycin and the AMPK inhibitor together (n = 2). The dotted line represents cells treated only with bafilomycin A1 (controls). Ctrl, control; phl, phloretin; baf, bafilomycin A1. Data are represented as mean ± s.e.m. *p < 0.05 and **p < 0.01

Since AMPK activation is related to the activation of catabolic processes in response to nutrient deprivation [33], we next investigated whether phloretin can activate autophagy. Autophagy is a conserved catabolic process that is induced upon starvation and other stress responses, which promotes the lysosomal degradation of intracellular cargo sequestered in vesicles termed autophagosomes. RNA-seq analysis predicted autophagy as one of the downstream biological processes showing increased activity in phloretin-treated BMDMs (z-score: 0.779) (Fig. 4A). Moreover, immunocytochemical analysis of BMDMs treated with phloretin and the autophagy inhibitor bafilomycin A1 displayed increased accumulation of the autophagy markers MAP1LC3/LC3 (microtubule-associated protein 1 light chain 3) and p62 compared to bafilomycin A1-treated BMDMs (Fig. 4B–D), indicating increased autophagic flux upon phloretin treatment [34]. Upon induction of autophagy, LC3 is converted from the LC3I form to the lipidated LC3II form, which correlates with the number of autophagosomes. In line with this, higher protein levels of LC3II and p62 were determined in phloretin-stimulated BMDMs by western blot (Fig. 4E, F). Interestingly, this increase in p62 and LC3II by phloretin was prevented by treating BMDMs with the AMPK inhibitor BML-275 (Fig. 4E–F). Collectively, our data show that phloretin stimulates autophagy in an AMPK-dependent manner.

### Phloretin activates the Nrf2 pathway through autophagy-mediated Keap1 degradation

**Fig. 5:**
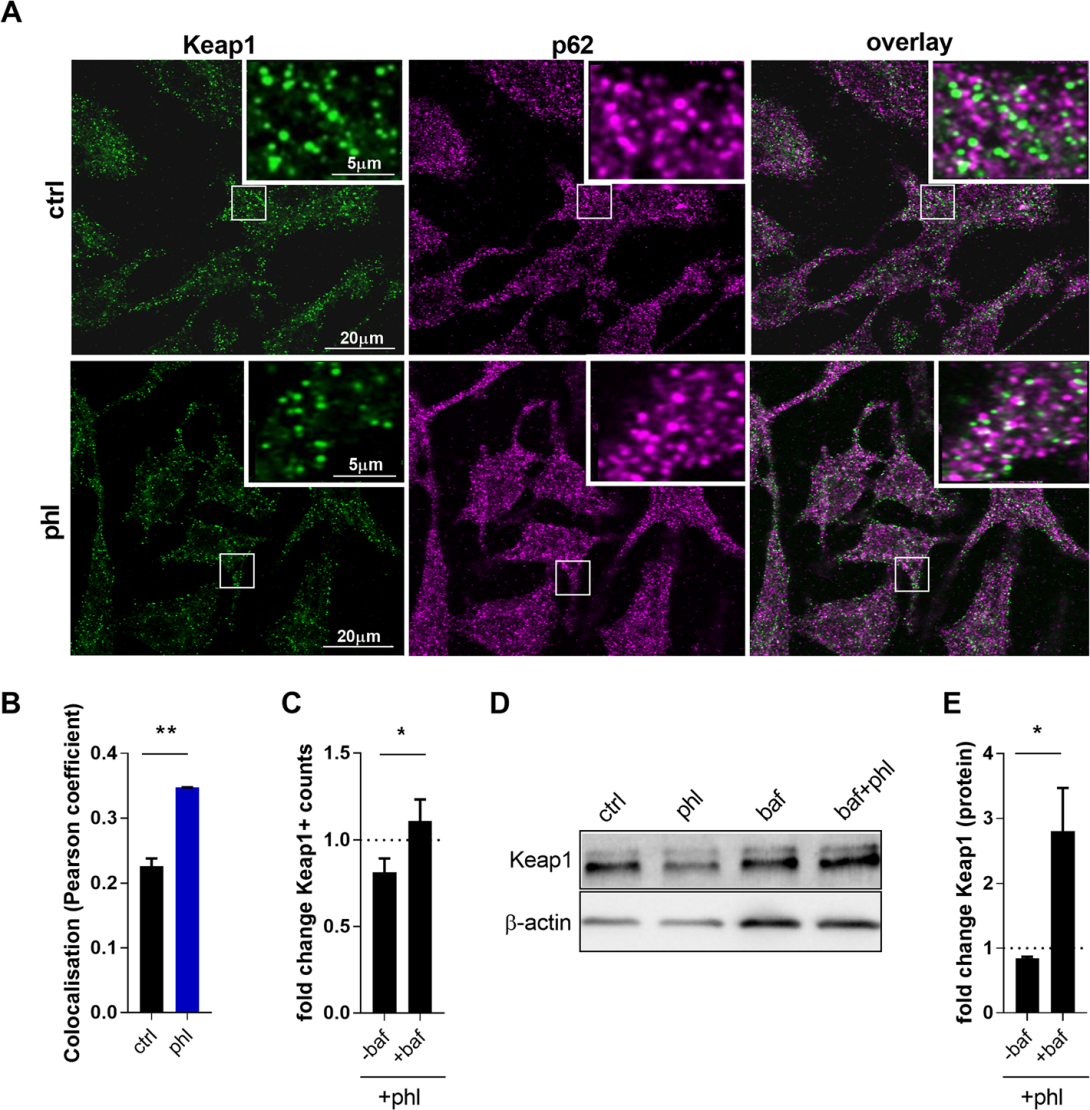
Phloretin activates the Nrf2 pathway through autophagy-mediated Keap1 degradation. **A**, **B** Representative images of Keap1 and p62 staining and quantification of their colocalization (Pearson’s coefficient) on control or phloretin-treated BMDMs (90+ cells per well, 3 wells). **C** Quantification of Keap1 positive counts in phloretin-treated BMDMs treated with or without bafilomycin A1 (90+ cells per well, 3 wells). The dotted line represents corresponding control cells stimulated with or without bafilomycin A1. **D**, **E** Western blot quantification and representative bands of phloretin-treated BMDMs treated with or without bafilomycin A1 (n = 4). The dotted line represents the corresponding control cells stimulated with or without bafilomycin A1. Ctrl, control; phl, phloretin; baf, bafilomycin A1. Data are represented as mean ± s.e.m. *p < 0.05 and **p < 0.01

Our data show that phloretin activates Nrf2 and stimulates autophagy in macrophages. Interestingly, the autophagy receptor p62 can compete with Nrf2 for binding to Keap1, the adaptor that facilitates Nrf2 ubiquitination and degradation under normal conditions. This p62-mediated dissociation of Keap1/Nrf2 will therefore prevent Nrf2 degradation and eventually lead to Nrf2 activation [35]. For this reason, we determined whether phloretin promotes p62/Keap1 interaction, thereby activating the Nrf2 pathway. Here, we show that phloretin activates the Nrf2 pathway through p62-mediated Keap1 degradation in macrophages. By using high-resolution Airyscan confocal microscopy combined with colocalization analysis, we show that phloretin stimulates the interaction of p62 and Keap1, as demonstrated by an increased value of the colocalization parameter (Pearson’s coefficient) in phloretin-treated BMDMs (Fig. 5A, B). Moreover, our findings show that lower protein levels of Keap1 are present in phloretin-treated BMDMs, confirming degradation of Keap1 (Fig. 5C). To confirm that the degradation is autophagy dependent, we added the autophagy inhibitor bafilomycin A1 to phloretin-treated BMDMs. Interestingly, this reduction in Keap1 was reversed by bafilomycin A1 treatment (Fig. 5C). These results were confirmed by western blot, showing a reduction in Keap1 protein levels in phloretin-treated BMDMs which was prevented by bafilomycin A1 (Fig. 5D, E).

### Phloretin reduces neuroinflammation in the EAE model

**Fig. 6:**
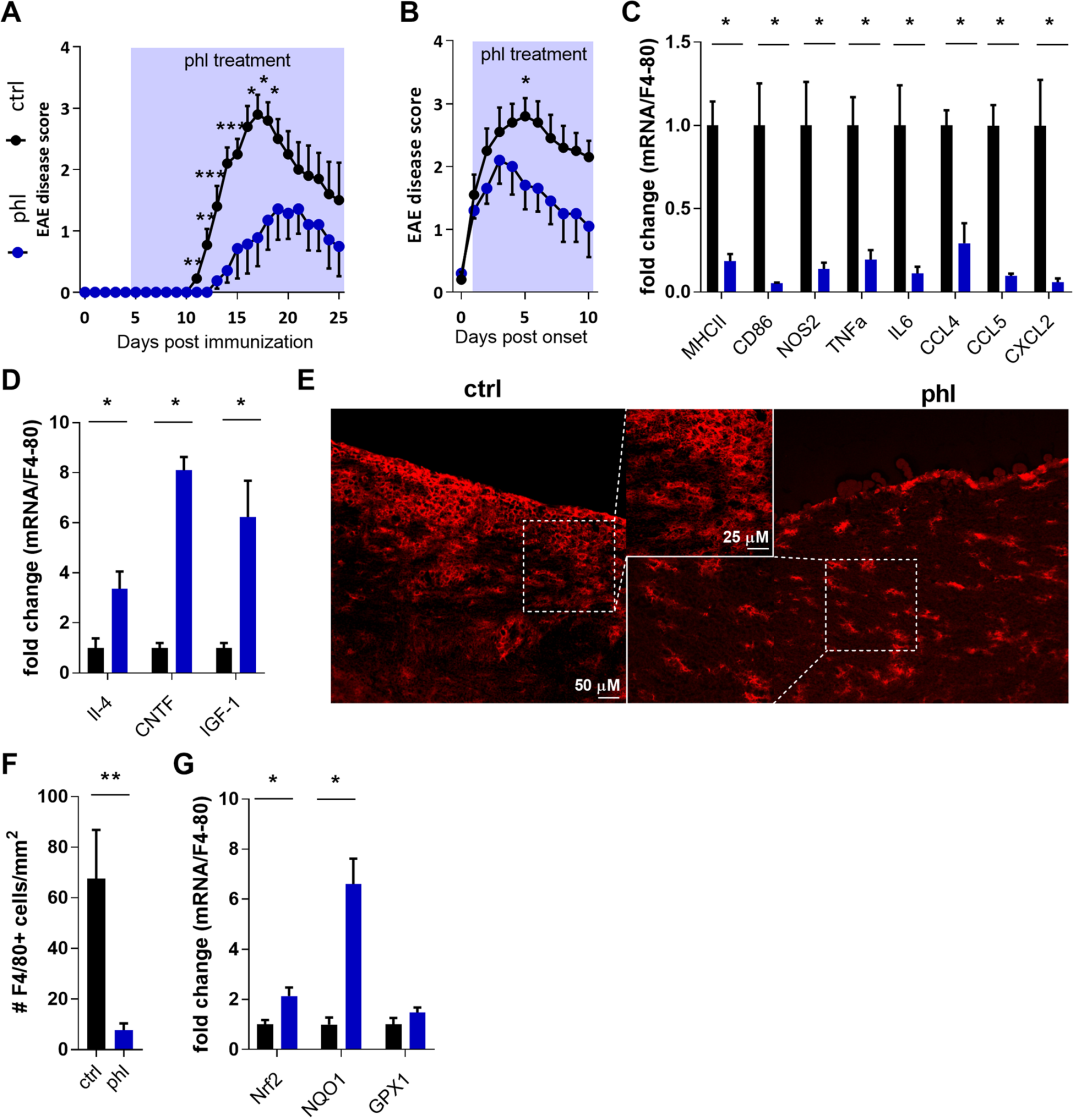
Phloretin reduces neuroinflammation in the EAE model. **A** Disease scores of EAE mice treated 6 days post immunization with vehicle or phloretin on a daily basis (prophylactic setting, 50 mg/kg ip, n = 5) **B** Disease scores of EAE mice treated with vehicle or phloretin on a daily basis after disease onset (disease score > 1) (therapeutic setup, 50 mg/kg ip, n = 5). **C**, **D** Quantitative PCR was used to determine the mRNA levels of the pro-inflammatory genes *MHCII*, *CD86*, *NOS2*, *TNFα*, *IL6*, *Ccl4*, *Ccl5* and *CXCL2* and the anti-inflammatory and neurotrophic genes *IL-4*, *CNTF* and *IGF-1* in the spinal cord of phloretin-treated EAE animals (prophylactic setting). **E**, **F** Quantification and representative images of F4/80 staining on spinal cord tissue obtained from EAE animals treated with vehicle or phloretin in the prophylactic setting. **G** Quantitative PCR was used to determine the mRNA levels of genes related to the Nrf2 pathway (*Nrf2*, *NQO1*, *GPX1*) in the spinal cord of phloretin-treated EAE animals (prophylactic setting). Gene expression was corrected for the number of F4/80+ cells. Ctrl, control; phl, phloretin. Data are represented as mean ± s.e.m. *p < 0.05 and **p < 0.01

To validate our findings in vivo, we investigated the impact of phloretin on the EAE model, the most commonly used animal model of MS, that is characterized by a pronounced macrophage-mediated inflammatory response in the CNS [5]. EAE animals treated with phloretin before disease onset showed reduced clinical scores compared to vehicle-treated animals (Fig. 6A). Importantly, even in a therapeutic setup, in which phloretin treatment was started after disease onset (clinical score > 1), phloretin reduced disease severity (Fig. 6B). Reduced disease severity in phloretin-treated animals was paralleled by a decreased expression of inflammatory genes *MHCII, CD86, Nos2*, *TNFα*, *IL-6*, *CCL4*, *CCL5* and *CXCL2* in the spinal cord (Fig. 6C). Moreover, a reduced amount of F4/80^+^cells was found in the spinal cord of phloretin-treated animals (Fig. 6E, F). Since both F4/80^+^ microglia and F4/80^+^ macrophages contribute to neuroinflammation, more in-depth analysis illustrated that phloretin markedly reduced the amount of F480+TMEM119− macrophages in EAE mice (supplementary Fig. 2 D-F) while a non-significant trend was observed towards a reduction in F480+TMEM119+ microglia. Consistent with this finding, phloretin suppressed the production of inflammatory mediators in microglia but this suppression was less pronounced compared to macrophages (supplementary Fig. 2 A-C). These findings suggest that EAE amelioration by phloretin is mainly macrophage-driven. In addition to reducing the expression of inflammatory genes, phloretin increased the expression of anti-inflammatory and neurotrophic factors, i.e. *IL-4*, *CNTF* and *IGF-1* in the spinal cord of EAE animals (Fig. 6D). These findings strongly suggest that phloretin reduces EAE disease severity by driving macrophages towards a disease-resolving phenotype. In line with our previous in vitro findings, we also detected elevated Nrf2 signalling in the CNS of phloretin-treated EAE animals as indicated by increased mRNA expression of *Nrf2* and it downstream targets *NQO1* and *GPX1* (Fig. 6G). Altogether, these data show that phloretin suppresses neuroinflammation in both a prophylactic and therapeutic setting. Furthermore, our findings indicate that phloretin may reduce neuroinflammation by suppressing the inflammatory features of macrophages through Nrf2 activation.

## Discussion

In this study, we demonstrate that phloretin drives macrophages towards a less-inflammatory phenotype in an Nrf2-dependent manner. AMPK-dependent activation of autophagy and subsequent Keap1 degradation was found to underlie Nrf2 activation by phloretin. Furthermore, we confirm the anti-inflammatory effects of phloretin in an EAE model where it decreases disease severity and alleviates macrophage-dependent neuroinflammation.

We show that phloretin suppresses the inflammatory phenotype of macrophages. This is in line with findings of Wei-Tien Chang et al. who demonstrated that phloretin has anti-inflammatory features in a macrophage cell line [36]. We demonstrate that the induction of this phenotype shift is dependent on Nrf2. Nrf2 is a master regulator of anti-oxidative responses and its activation is known to drive macrophages towards an anti-inflammatory phenotype [11, 12, 37]. In addition, several studies defined that Nrf2 activation attenuates neuroinflammation and neurodegeneration in CNS disorders [38–40]. However, although our findings show that Nrf2 underlies the phenotype shift of phloretin-treated macrophages, RNA sequencing data illustrated that, amongst Nrf2 activation, other pathways were upregulated. In particular, the upregulation of the PPAR pathway is of interest due to its anti-inflammatory features and crosstalk with Nrf2 [41–45]. Additionally, since phloretin is a well-defined GLUT inhibitor and switching to glycolysis is a crucial metabolic event for macrophage activation, phenotype changes might also be partly induced by direct metabolic effects of phloretin on these glucose transporters [46, 47]. More research is warranted to define the relevance of PPAR in phloretin-induced Nrf2 activation and to what degree phloretin-induced immunomodulation is influenced by direct metabolic changes. Overall, our findings clearly demonstrate that Nrf2 activation plays an essential role in the phloretin-mediated phenotype switch.

Our findings indicate that Nrf2 activation by phloretin is mediated by AMPK activation. AMPK plays a crucial role in restoring cellular energy homeostasis in the case of stresses that deplete ATP resulting in increased ADP:ATP ratio, thereby switching on alternate catabolic pathways that generate ATP, while turning off anabolic pathways that consume ATP [48]. Correspondingly, phloretin is a well-established GLUT inhibitor and lowers cellular glucose levels [49–51]. Several studies demonstrated that AMPK activation in macrophages induces an immunosuppressive phenotype, which corroborates our results showing that AMPK activation is essential for phloretin to lower inflammation [52, 53]. Our data are also consistent with previous studies wherein phloretin has been demonstrated to activate AMPK in other cell types including adipocytes and endothelial cells [50, 54–56]. Phloretin may activate AMPK indirectly by increasing AMP and lowering ATP levels, since AMP and ATP allosterically stimulate or inhibit AMPK activation, respectively [48, 57]. Nevertheless, CaMKKβ, which is a kinase upstream AMPK, can promote AMPK activation in response to increased cellular Ca^2+^ levels without any change in AMP/ATP levels [58]. In addition, given the wide network of the interplay between AMPK and up- and downstream pathways, further research is needed to elucidate the underlying mechanism of phloretin-induced AMPK activation.

Our data suggest that phloretin-mediated AMPK activation stimulates autophagy in macrophages. As mentioned above, AMPK activation is a self-protective process that aims to restore the energy balance of the cell [48]. Although no study has ever shown that phloretin was able to stimulate the phenotype of macrophages by promoting autophagy, some studies have nevertheless demonstrated an effect of phloretin on autophagy pathways in cancer cells and hepatocytes [59–61]. Moreover, various studies show that downstream catabolic processes of AMPK activation can activate autophagy [60, 62, 63]. Interestingly, in response to glucose starvation, AMPK-mediated autophagy is induced by phosphorylation of the important autophagy initiator unc-51 like autophagy activating kinase [64, 65]. With respect to this, we speculate that AMPK stimulates autophagy to restore ATP from cellular components in response to phloretin-induced glucose depletion. However, glucose starvation can also activate autophagy in an AMPK-independent manner [66–68]. Therefore, more research is necessary to determine whether autophagy activation by phloretin also occurs partially independently of AMPK.

Recent studies showed the importance of autophagy to drive the functional macrophage phenotype. In this context, dysregulation of autophagy in macrophages has notably been linked to the onset of atherosclerosis and neurological diseases [69–72]. Here, we provide a link between phloretin treatment, autophagy induction, and Nrf2 activation by showing that phloretin promotes Nrf2 activation via p62-mediated degradation of Keap1. Until recently, an increase in p62 puncta was regarded as a sign of decreased autophagy as p62 is degraded in autophagosomes upon autophagy activation [34]. In relation to this, p62 accumulation has been linked to autophagy dysfunction in atherosclerotic lesions [73]. However, this notion has been challenged in recent years and it is now thought that p62 is necessary for the formation of autophagosomes and that an increase in p62 levels in parallel with an increase in LC3II puncta can be a sign of autophagy induction [74]. Therefore, together with the capacity of phloretin to activate AMPK and its known role in autophagy activation, our results suggest that the P62 and LC3 accumulation upon bafilomycin A1 treatment in phloretin-stimulated macrophages is due to increased autophagic flux and not due to impaired autophagy. Interestingly, Lee Y et al. recently showed that, besides activating Nrf2, p62 binding to Keap1 is also involved in autophagosome formation, suggesting that phloretin-mediated autophagy activation is directly linked to increased p62 activity on one hand, but also to more Keap1-p62 interaction on the other hand [75]. Keap1 is an adaptor of Cullin-3-based ubiquitin ligase that forms a homodimer with Nrf2 at homeostatic conditions, thereby promoting Nrf2 ubiquitination and proteasomal degradation [76]. Disruption of the Keap1-Nrf2 complex leads to Nrf2 stabilization and translocation to the nucleus [77, 78]. It is well documented that disruption of this complex occurs via either modification of Keap1 cysteine residues by electrophilic molecules, direct interaction of small molecules with Keap1, or p62-mediated Keap1 degradation [35]. Ying Y et al. suggest a direct interaction of phloretin and Keap1 based on in silico experiments, which then leads to activation of the Nrf2 pathway in a cardiomyocyte cell line [79]. Here, we established an additional autophagy-related mechanism by which phloretin activates Nrf2.

By using the EAE model, we established that phloretin alleviates neuroinflammation in vivo. Our data strongly suggest that phloretin ameliorates EAE by suppressing the inflammatory response mediated by macrophages and activating the Nrf2 pathway. In other disease models, phloretin was also reported to exert neuroprotective and immune-modulatory features. In particular, phloretin was found to activate the Nrf2 pathway in the brain upon cerebral ischemia and decrease amyloid beta accumulation in the rat Alzheimer’s disease model [80–84]. Phloretin might also affect other immune cells in vivo, as this compound has been found to inhibit T cell and dendritic cell activation in vitro [85, 86]. Given that neuroinflammation in the EAE model is not purely macrophage dependent, it would be of interest to define to what extent the reduction in neuroinflammation is mediated by other immune cells. With respect to this, although our data suggests that EAE amelioration is mainly macrophages driven and is less dependent on microglia modulation, further experiments are needed to elucidate to what extent phloretin affects microglia in neuroinflammatory diseases. Overall, our findings demonstrate that phloretin reduces neuroinflammation by affecting the phenotype of macrophages, showing the therapeutic potential of phloretin for neuroinflammatory disorders.

## Conclusions

Our study demonstrates that phloretin is a potent immunomodulatory agent that modulates the inflammatory properties of macrophages in neuroinflammatory disorders. Activation of the Nrf2 pathway, mediated by AMPK-dependent autophagy activation and subsequent Keap1 degradation, was established to drive this phenotype shift. Hence, phloretin is a promising naturally occurring agent that can be used to lower the inflammatory burden of neuroinflammatory diseases such as MS.

## Supplementary Information

**Supplementary Figure 1 A.** Volcano plot showing that upon phloretin treatment in activated macrophages the expression of 223 genes was upregulated while that of 215 genes was downregulated. Differentially expressed genes were used as input for the core analysis in ingenuity pathway analysis (IPA) (*n*=5, cut-off criteria *p*<0.05). Dots representing differentially expressed genes associated to pro-inflammatory canonical pathways are appointed by name. **B.** Volcano plot demonstrating that phloretin treatment in macrophages increased the expression of 355 genes while decreasing that of 288 genes (*n*=5, cut-off criteria *p*<0.05). Differentially expressed genes associated to the Nrf2 pathway pathways are appointed by name.

**Supplementary Figure 2 A.** ROS production in vehicle- or phloretin-treated microglia stimulated with PMA (3 independent experiments, *n*=19 wells). **B.** NO production in vehicle- or phloretin-treated microglia stimulated with LPS (3 independent experiments, *n*=19-21 wells) **C.** mRNA levels of the pro-inflammatory genes *IL-6, NOS2, COX2 and IL-12* in vehicle- or phloretin-treated microglia stimulated with LPS (3 independent experiments, n=16-17 wells). **D-F.** Quantification and representative images of F4/80^+^TMEM119^-^ macrophages and F4/80^+^TMEM119^+^ microglia on spinal cord tissue obtained from EAE animals treated with vehicle or phloretin in the prophylactic setting. Ctrl, control; phl, phloretin. Data are represented as mean ± s.e.m. *p < 0.05, **p < 0.01 and ***p < 0.001

## Data Availability

The datasets used and/or analysed during the current study are available from the corresponding author on reasonable request.

## Abbreviations

AMPK: AMP-activated protein kinase
BMDMs: Bone marrow-derived macrophages
CNS: Central nervous system
EAE: Experimental autoimmune encephalomyelitis
Fc: Fold change
FCS: Foetal calf serum
GLUT: Glucose transporter
IPA: Ingenuity pathway analysis
Keap1: Kelch-like ECH-associated protein 1
KO: Knockout
LCM: L929-conditioned medium
LPS: Lipopolysaccharide
MAP1LC3/LC3: Microtubule-associated protein 1 light chain 3
MS: Multiple sclerosis
NO: Nitric oxide
Nrf2: Nuclear factor erythroid 2–related factor 2
PMA: Phorbol 12-myristate 13-acetate
RNA-seq: RNA sequencing
ROS: Reactive oxygen species
RT: Room temperature
RT-qPCR: Quantitative reverse transcription PCR
TBS-T: Tween-20
WT: Wild-type
