# Supplementary figures and images for: Phloretin suppresses neuroinflammation by autophagy-mediated Nrf2 activation in macrophages
Source: J Neuroinflammation. 2021 Jul 4;18:148. doi: 10.1186/s12974-021-02194-z (PMC8254976; doi:10.1186/s12974-021-02194-z)

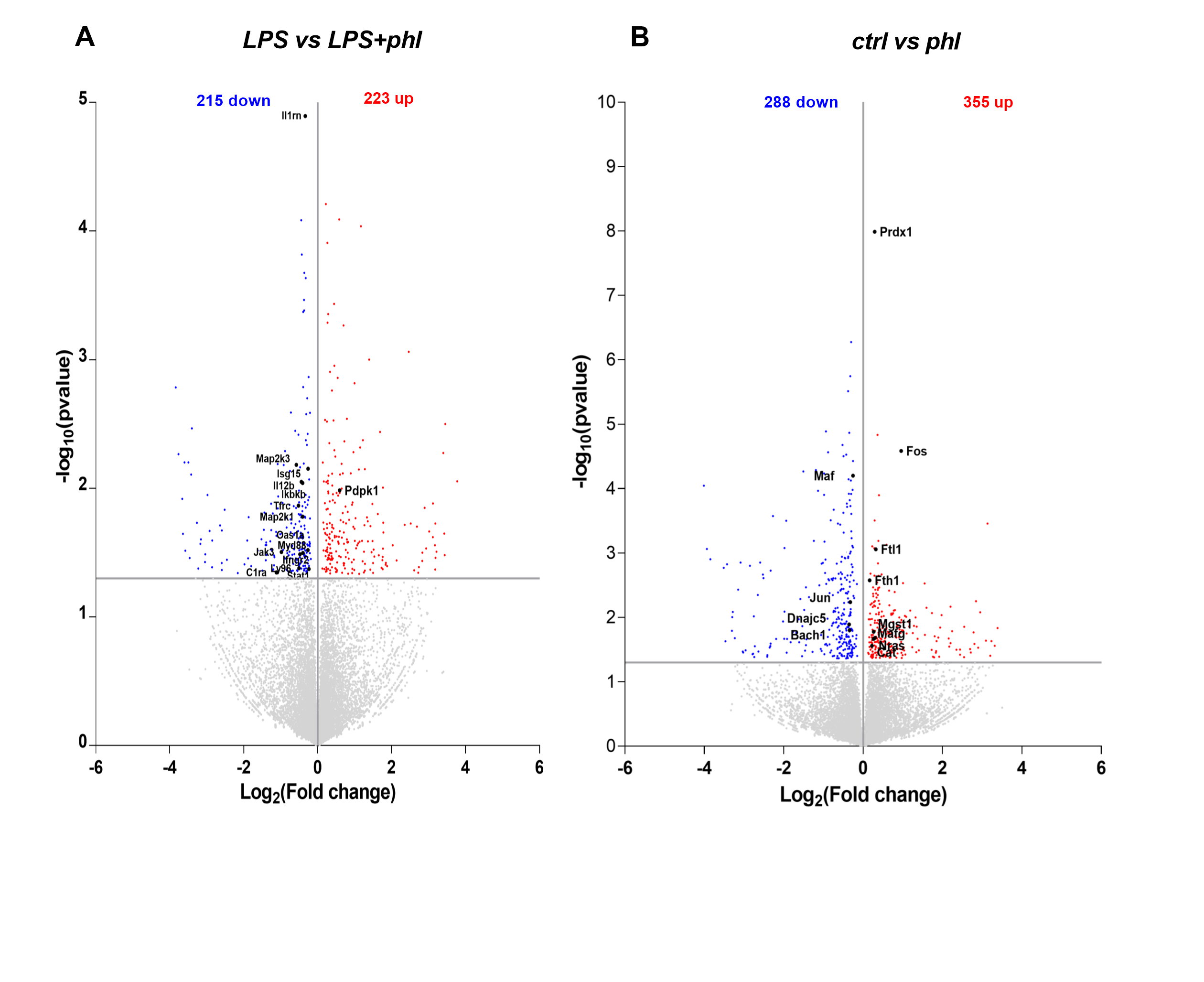

Supplement: Supplementary file 1 — Supplementary Figure 1 A. Volcano plot showing that upon phloretin treatment in activated macrophages the expression of 223 genes was upregulated while that of 215 genes was downregulated. Differentially expressed genes were used as input for the core analysis in ingenuity pathway analysis (IPA) (n=5, cut-off criteria p<0.05). Dots representing differentially expressed genes associated to pro-inflammatory canonical pathways are appointed by name. B. Volcano plot demonstrating that phloretin treatment in macrophages increased the expression of 355 genes while decreasing that of 288 genes (n=5, cut-off criteria p<0.05). Differentially expressed genes associated to the Nrf2 pathway pathways are appointed by name. [file 12974_2021_2194_MOESM1_ESM.tif]

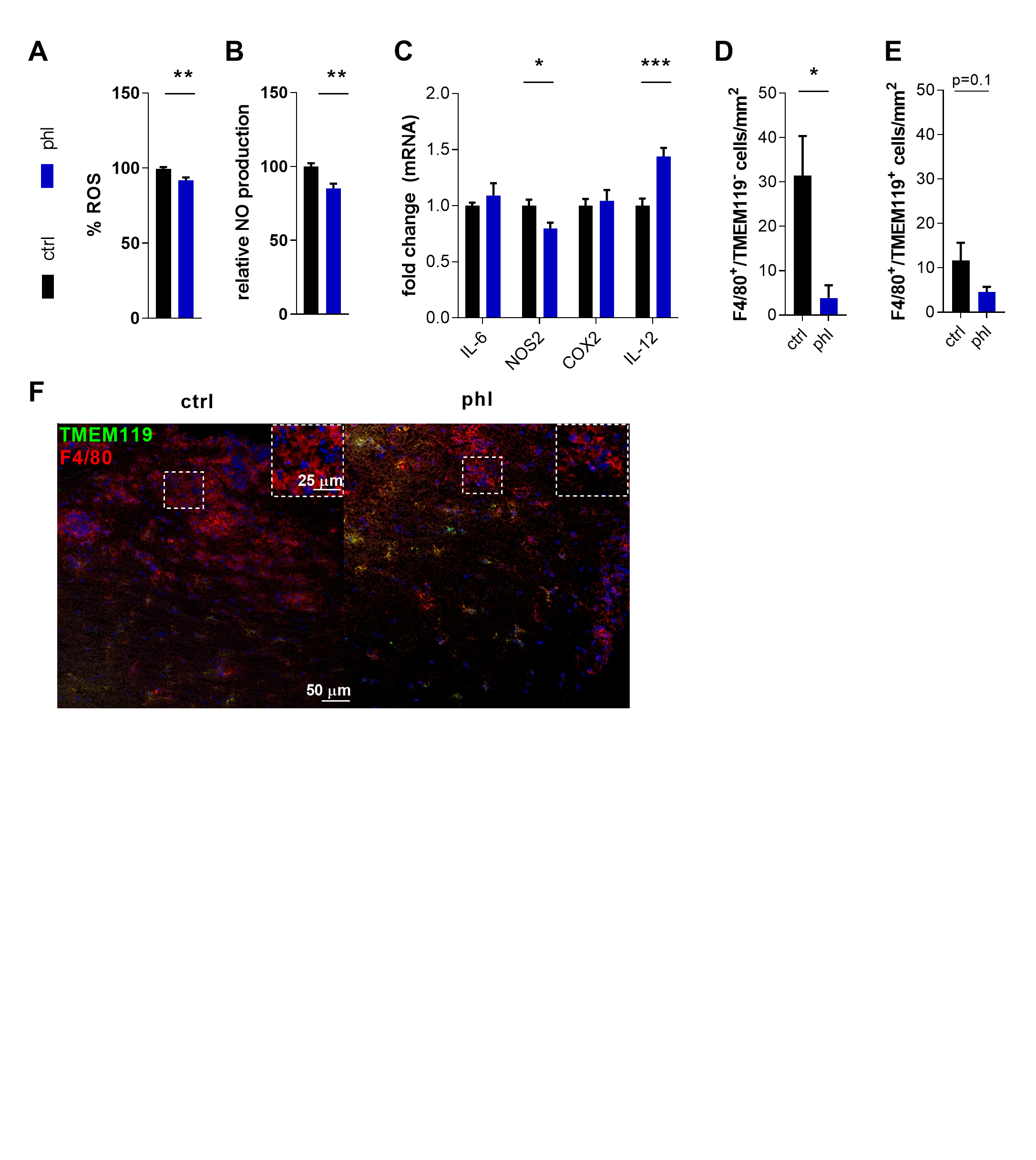

Supplement: Supplementary file 2 — Supplementary Figure 2 A. ROS production in vehicle- or phloretin-treated microglia stimulated with PMA (3 independent experiments, n=19 wells). B. NO production in vehicle- or phloretin-treated microglia stimulated with LPS (3 independent experiments, n=19-21 wells) C. mRNA levels of the pro-inflammatory genes IL-6, NOS2, COX2 and IL-12 in vehicle- or phloretin-treated microglia stimulated with LPS (3 independent experiments, n=16-17 wells). D-F. Quantification and representative images of F4/80+TMEM119- macrophages and F4/80+TMEM119+ microglia on spinal cord tissue obtained from EAE animals treated with vehicle or phloretin in the prophylactic setting. Ctrl, control; phl, phloretin. Data are represented as mean ± s.e.m. *p < 0.05, **p < 0.01 and ***p < 0.001 [file 12974_2021_2194_MOESM2_ESM.tif]
